# The forewing of the *Aphis fabae* (Scopoli 1763) (Hemiptera, Sternorrhyncha): a morphological and histological study

**DOI:** 10.1007/s00435-017-0358-7

**Published:** 2017-05-19

**Authors:** Barbara Franielczyk-Pietyra, Piotr Wegierek

**Affiliations:** Department of Zoology, Faculty of Biology and Environmental Protection, University of Silesia, Bankowa 9, 40-007 Katowice, Poland

**Keywords:** Aphids, Cross-section, Forewing, Tracheae, Wing veins

## Abstract

Dorsal and ventral sides of the forewing of *Aphis fabae* (Scopoli 1763) (Sternorrhyncha, Hemiptera) were examined by scanning electron microscopy. Reinforcement elements on their surface as well as scale-like elements were described. Using histological methods, cross-sections of the material were done. They showed a two-layered membrane with a circular foramen inside. The course of veins and places of their bifurcation were followed. Common stem of radius (R), media (M), and cubitus anterior (CuA) veins were composed of separate tracheae, which ran separately at the beginning, then continued in a single tunnel, and finally disappeared. Nerves were not observed. Neither were tracheae visible on the further course of those veins. The presence of a devoid-of-trachea costal vein was confirmed. Under scanning electron microscope, convex reinforcements on dorsal side of the wing turned out to be more sclerotized parts of chitin, not giving a zigzag-like profile of the wing on sections. In this paper, we show for the first time a cross-section of a very delicate wing of an aphid representative.

## Introduction

Insect body must be resistant to many environmental factors. It is especially true in case of wings, particularly in long-distance flying insects (Dirks and Taylor 2012). An insect wing is a two-layer membrane supported by longitudinal veins, sometimes also cross-veins and reinforced by extracellular cuticle. Wing veins are described as hollows circular on section, providing hemolymph, nerves and tracheae (Kukalová-Peck 1978; Dudley 2000; Shimmi et al. 2014). Tracheae appear in the wing before veins so it seems that they determine the course of the veins. However, not every vein comprises trachea (Patch 1909). Likewise, nerves or hemolymph are not always present in veins. Also, vein sections can be far from circular—from oval to campanulate (Snodgrass 1935; Dudley 2000), but most popular are dumbbell-shaped (Rajabi et al. 2016b).

Around veins thickened cuticle creates reinforcements, usually depicted as convex (above wing membrane as cuticular ridges in dorsal view) or concave (as cuticular grooves in ventral view), which cause wings corrugation (Kukalová-Peck 1978; Appel et al. 2015). Until now, it has been believed that only veins strengthen the wings, but actually it is achieved thanks to the interaction of veins, wing membrane, and corrugations (Rajabi et al. 2016a).

The second essential element relevant to flight is the wing base articulation (Chapman 2013). It is a very complex part of insect body, composed of axillary sclerites and additional elements working together during the flight and wing folding. Because axillary sclerites are tiny, it is easier to study larger insects, such as Odonata (Ninomiya and Yoshizawa 2009) or Dictyoptera (Yoshizawa 2011) in regard to Hemimetabola. Within the hemimetabolous suborder Sternorrhyncha (Hemiptera), wing base articulation was studied by Weber (1928, 1935) in aphids and whiteflies, Koteja (1996) in coccids, Yoshizawa and Saigusa (2001) and Ouvrard et al. (2008) in psyllids. More recently, forewing base articulation in representatives of all four infraorders of Sternorrhyncha was studied by Franielczyk and Wegierek (2016).

The presence of tracheae in Sternorrhyncha wings was studied in detail by Patch (1909), where she tried to homologize nymphal trachea with veins of imago. Tracheal system and veins pattern were also established for *Orthezia urticae* (Sternorrhyncha) by Koteja (1986). Because hemipteran wings are very small and fragile, latter one is the only article showing drawing of their cross-sections. The course of aphid wing veins was discussed by Klimaszewski and Wojciechowski (1992) and Wojciechowski (1992) for both fossil and recent groups and by Shcherbakov (2007), Szwedo et al. (2015) for fossil species.

A few other studies were recently carried out on wings of Orthoptera (Wootton et al. 2000), Lepidoptera (O`Hara and Palazotto 2012), Coleoptera (Sun et al. 2014), and Odonata (Appel et al. 2015; Rajabi et al. 2016a, b), whose wings are bigger and more rigid than those of Sternorrhyncha.

Here, we present the first reconstruction of the course of wing veins in aphids, which is also the first one within the Sternorrhyncha group.

In this study, we investigated dorsal and ventral surfaces of *Aphis fabae* (Scopoli 1763) forewing. Cross-sections of this forewing were made to find out what the inner structure looks like and to follow the course of the veins.

## Materials and methods

### Scanning electron microscopy

Forewings of three individuals of *Aphis fabae* species were examined using scanning electron microscopy. Samples were fixed and stored in 70% ethanol and then prepared using ethanol dehydration and hexamethyldisilazane (HMDS) drying. After 70% ethanol fixation, the material was dehydrated in a graded ethanol/water series of 75, 80, 90, 96, and 100% for 10 min in each concentration, and then there were three 100% ethanol changes. After dehydration, the samples were treated with HMDS 3 × 10 min and retained in HMDS after third change until the solution evaporated (Kanturski et al. 2015).

Samples were mounted on holders, sputter-coated with gold and examined using a scanning electron microscope (Hitachi UHR FE-SEM SU 8010, Tokyo, Japan) in the Scanning Electron Microscopy Laboratory at the Faculty of Biology and Environmental Protection, University of Silesia.

### Histology

Specimens were collected in 70% ethanol and then transferred to 2.5% glutaraldehyde in a 0.05 M cacodylate buffer (pH 7.4). After washing in 0.1 M phosphate buffer (pH 7.4), the material was postfixed for 2 h using 1% OsO_4_ in phosphate buffer, dehydrated in a graded series of ethanol replaced by acetone and then embedded in an Epoxy Embedding Medium Kit (Sigma, St. Louis, MO). Semithin sections were cut from the root to the tip of the forewing on a Leica Ultracut UCT ultramicrotome (each having a thickness of 700 nm) with diamond knife and stained with methylene blue.

**Fig. 1:**
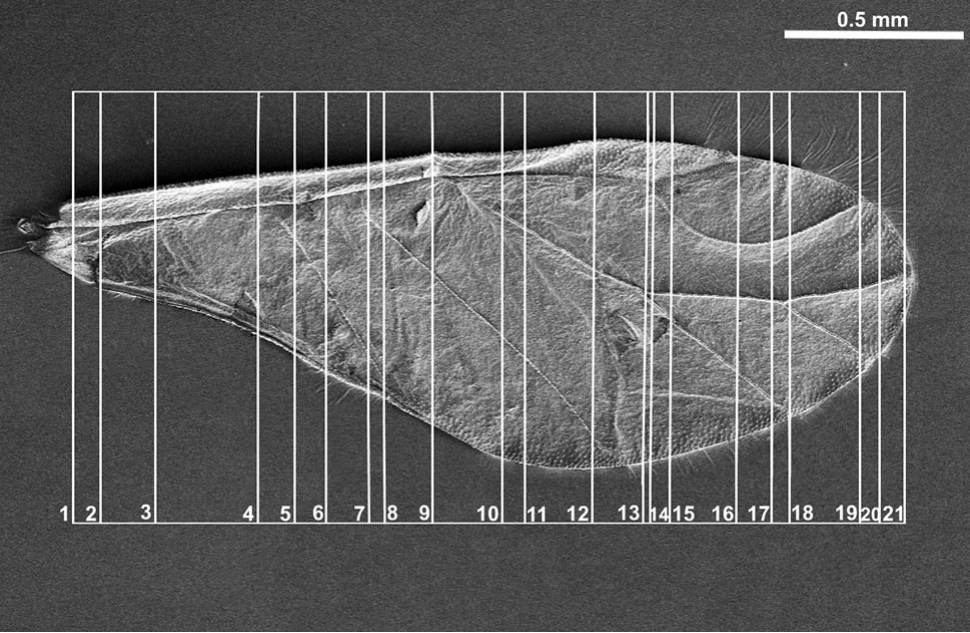
Scanning electron microscopy showing the forewing of *Aphis fabae* (Scopoli 1763), places of sectional cuts

Sectional cuts (Fig. 1) were analyzed using Nikon Ni-U light microscope and photographed with a Nikon DS-Fi2 camera. The whole wing was cut into about 600 semithin sections but 21 slices were selected. They are aligned in Figs. 6, 7, 8, and 9 the same way as the white lines on SEM images (costal margin at the top, anal margin at the bottom, upper surface to the left). Some of the slices were positioned at an angle to use the available space efficiently.

Facing the problem of non-consistent nomenclature of the veins, we adopted that of Shcherbakov (2007), Szwedo and Nel (2011), and Nel et al. (2012). The following abbreviations are used: C—costa; R1 (=RA)—radius (=radius anterior); Rs (=RP)—radius sector (=radius posterior); M—media; M_1_—first branch of media; M_2_—second branch of media; M_3+4_—fused third and fourth branch of media; CuA_1_—first branch of cubitus anterior; CuA_2_—second branch of cubitus anterior.

## Results

### Surface of the forewing

Dorsal surface of *Aphis fabae* forewing is characterized by strongly convex reinforcement elements (Figs. 3a, 4a, 5; white arrows), each one covered by ring-like elements (additional support in the form of rings arranged one after another). Almost entire edge of the wing is covered by scale-like elements (Figs. 3, 4, 5a, b; white asterisk). However, the dorsal side is not as much covered by wax as the ventral one. What is more, on the surface of the latter all reinforcement elements are concave (Figs. 3b, 4b).

Pterostigma on both sides, the dorsal and ventral sides, is covered by scale-like elements with denser arrangement dorsally (Fig. 3a, b; white asterisk).

**Fig. 2:**
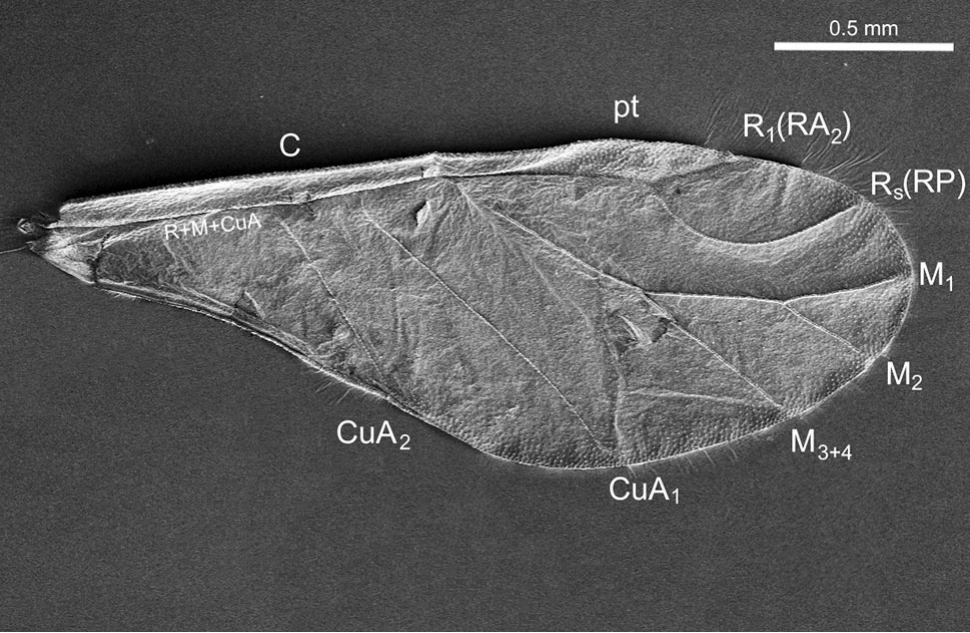
Scanning electron microscopy showing the forewing of *Aphis fabae* (Scopoli 1763), veins organization, dorsal view

**Fig. 3:**
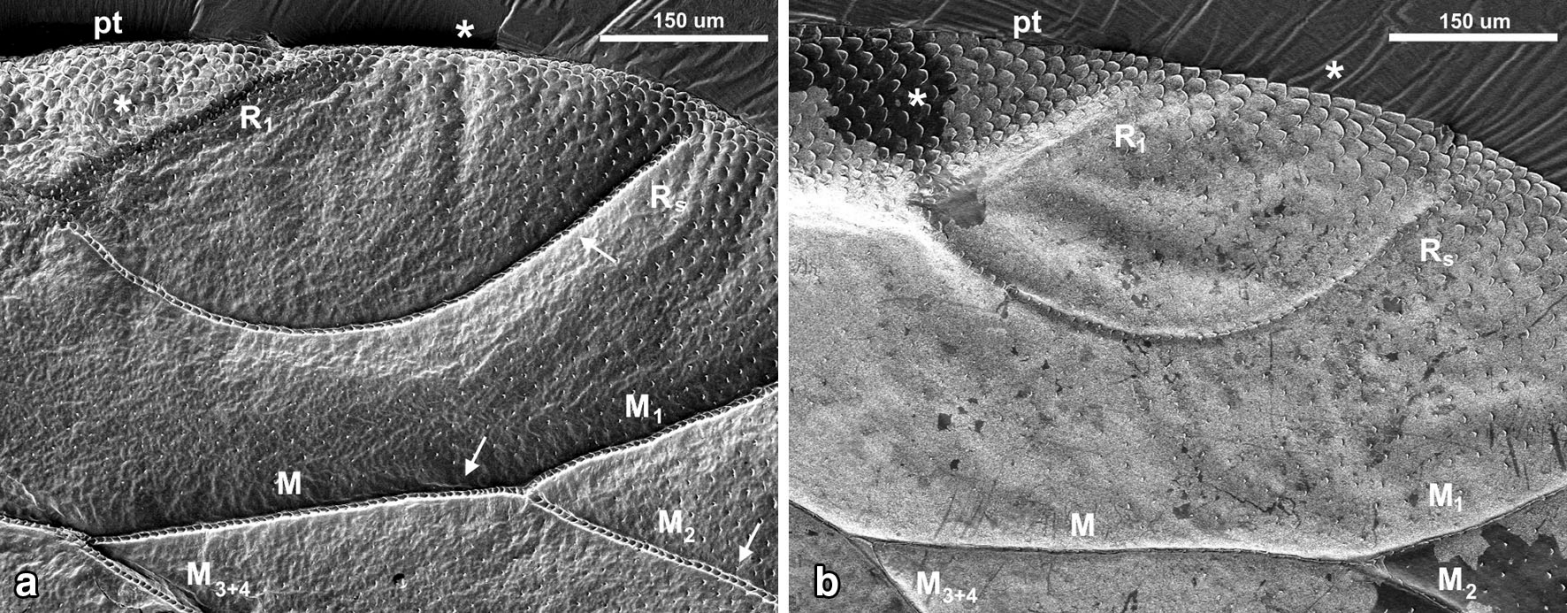
Scanning electron microscopy showing part of the wing of *Aphis fabae* (Scopoli 1763), with pterostigma (pt) **a** dorsal view, **b** ventral view; *white arrows* indicate reinforcement elements; *white asterisks* indicate scale-like elements

**Fig. 4:**
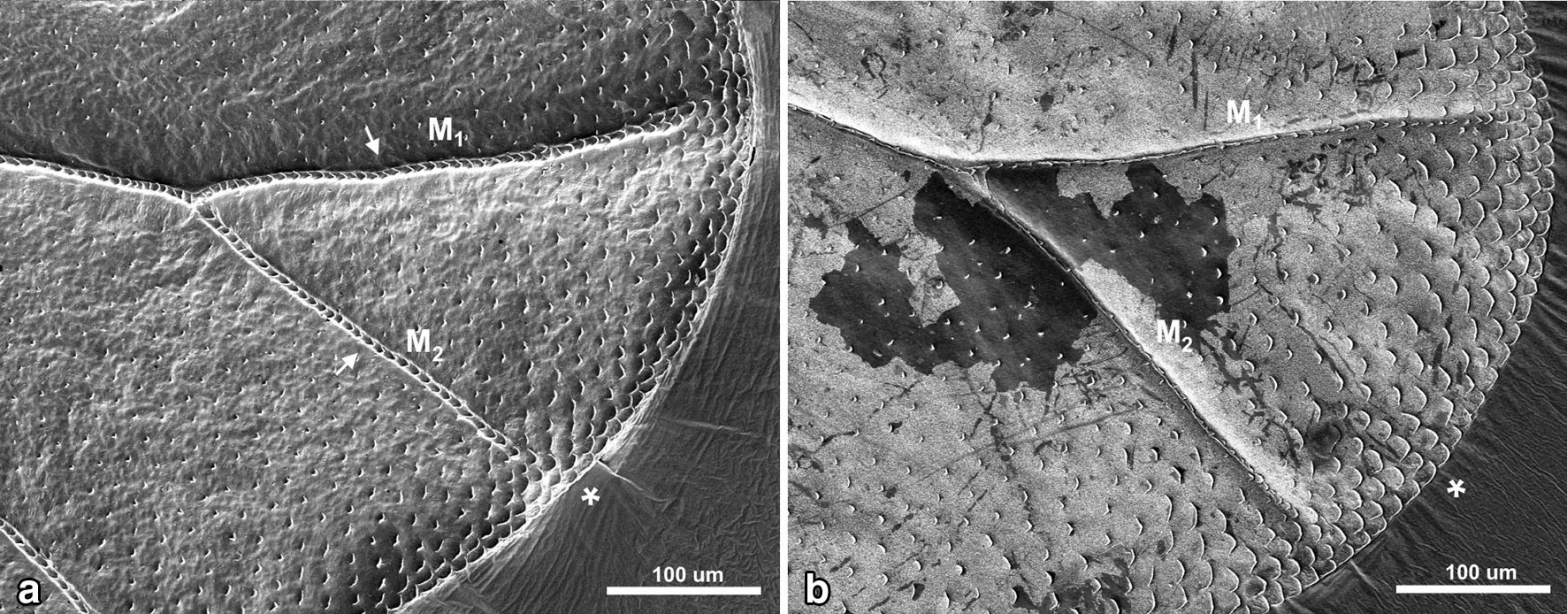
Scanning electron microscopy showing apical part of the wing of *Aphis fabae* (Scopoli 1763) **a** dorsal view, **b** ventral view, *white arrows* indicate reinforcement elements; *white asterisks* indicate scale-like elements

**Fig. 5:**
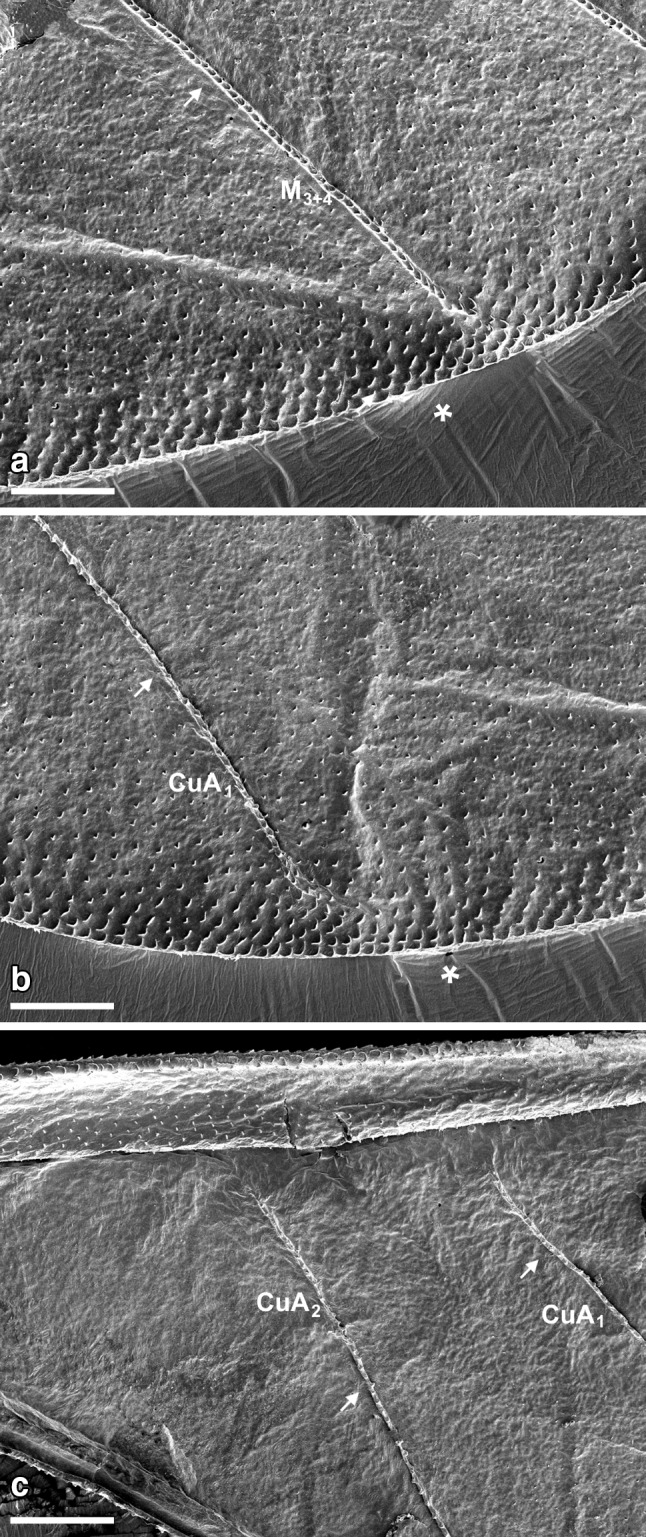
Scanning electron microscopy showing the wing of *Aphis fabae* (Scopoli 1763) **a** apical part of media vein (M_3+4_), dorsal view, **b** apical part of first branch of cubitus vein (CuA_1_), dorsal view, **c** part of common stem and both branches of cubitus vein (CuA_1_, CuA_2_), dorsal view; *white arrows* indicate reinforcement elements; *white asterisks* indicate scale-like elements; *scale bar* 100 µm

All reinforcement elements are convex on the dorsal side and concave ventrally (Figs. 3, 4). Along the cephalic margin of the wing the costal vein (C) extends to the region of pterostigma (Fig. 2). Parallel to C, common stem is visible, composed of radial (R), media (M), and cubitus anterior (CuA) veins (R + M + CuA). Radial vein is divided into R1 and Rs, right behind pterostigma. Medial vein consists of M_1_, M_2,_ and M_3+4_. Cubitus anterior is divided into two branches, CuA_1_ and CuA_2_. Anal veins and claval fold are absent. There is no direct connection between the so-called common stem and each vein (Fig. 5c). Also, none of the mentioned veins reach the apical part of the wing directly (Figs. 3, 4, 5a, b) and there are no cross-veins.

### Cross-sections of the forewing

All veins are recognized on sections and all are rounded, but only costal vein is clearly round in shape. Other veins are almost round.

On sections, veins are arranged in one straight line, there is no zigzag-like profile. Only more sclerotized cuticle around veins is marked as dark points on cross-sections (as around CuA_2_ in Fig. 7a).

**Fig. 6:**
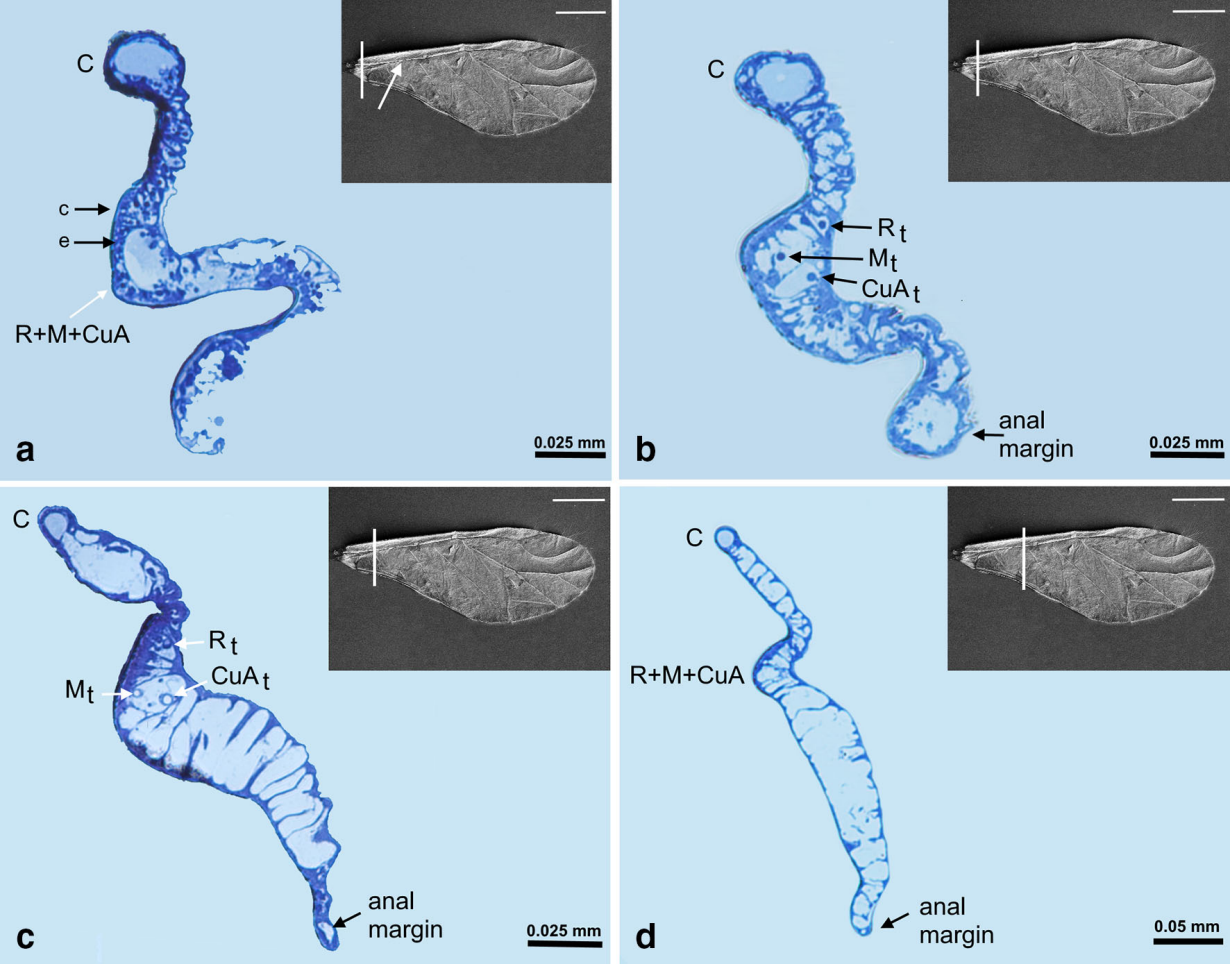
Cross-sections of the forewing of *Aphis fabae* (Scopoli 1763) under magnification **a**–**c** ×40, **d** ×20; *LM* light microscope, *c* cuticle, *e* epidermal cells. SEM *scale bar* 0.5 mm

**Fig. 7:**
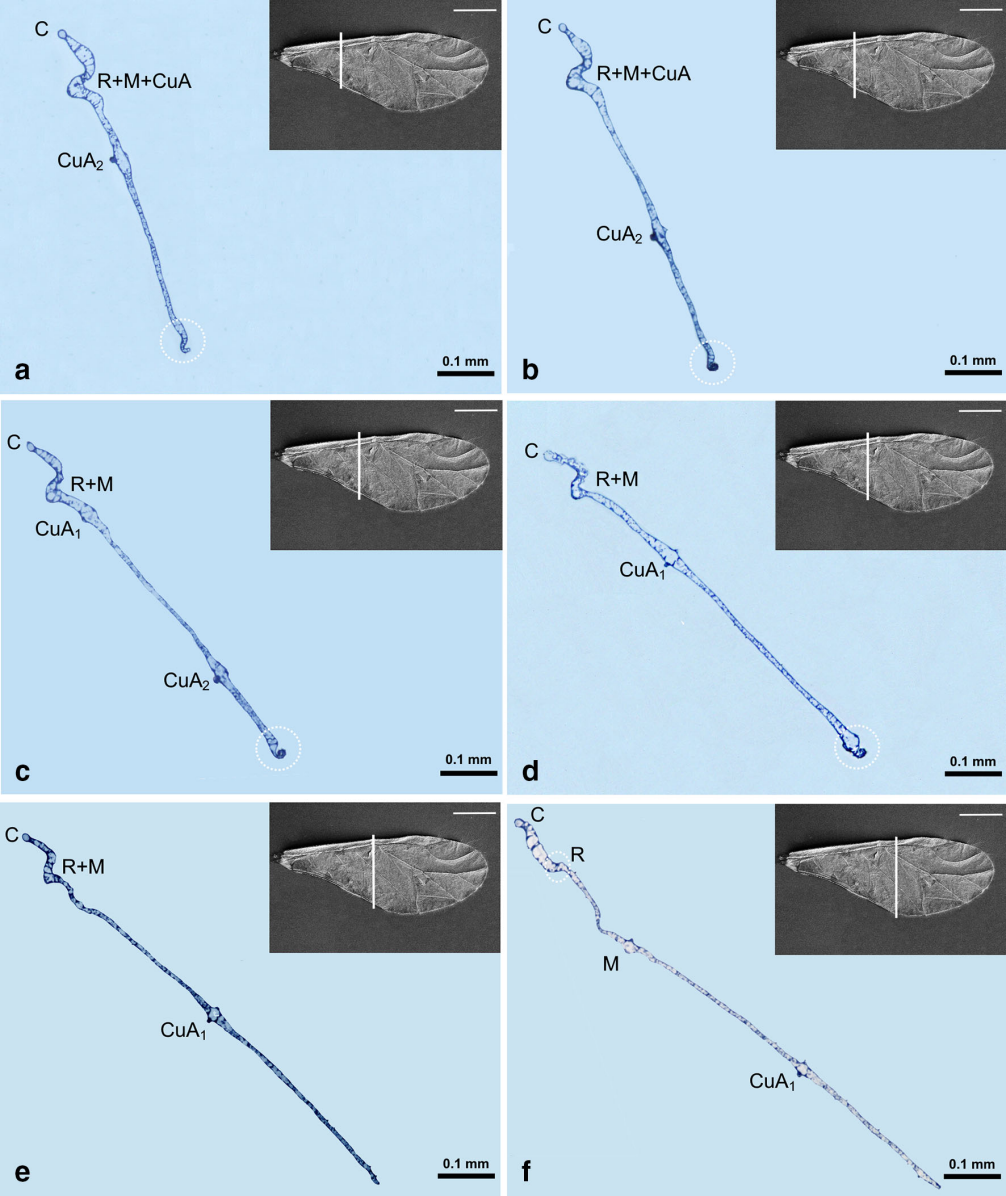
Cross-sections of the forewing of *Aphis fabae* (Scopoli 1763) under magnification **a**–**f** ×10; LM. SEM *scale bar* 0.5 mm

**Fig. 8:**
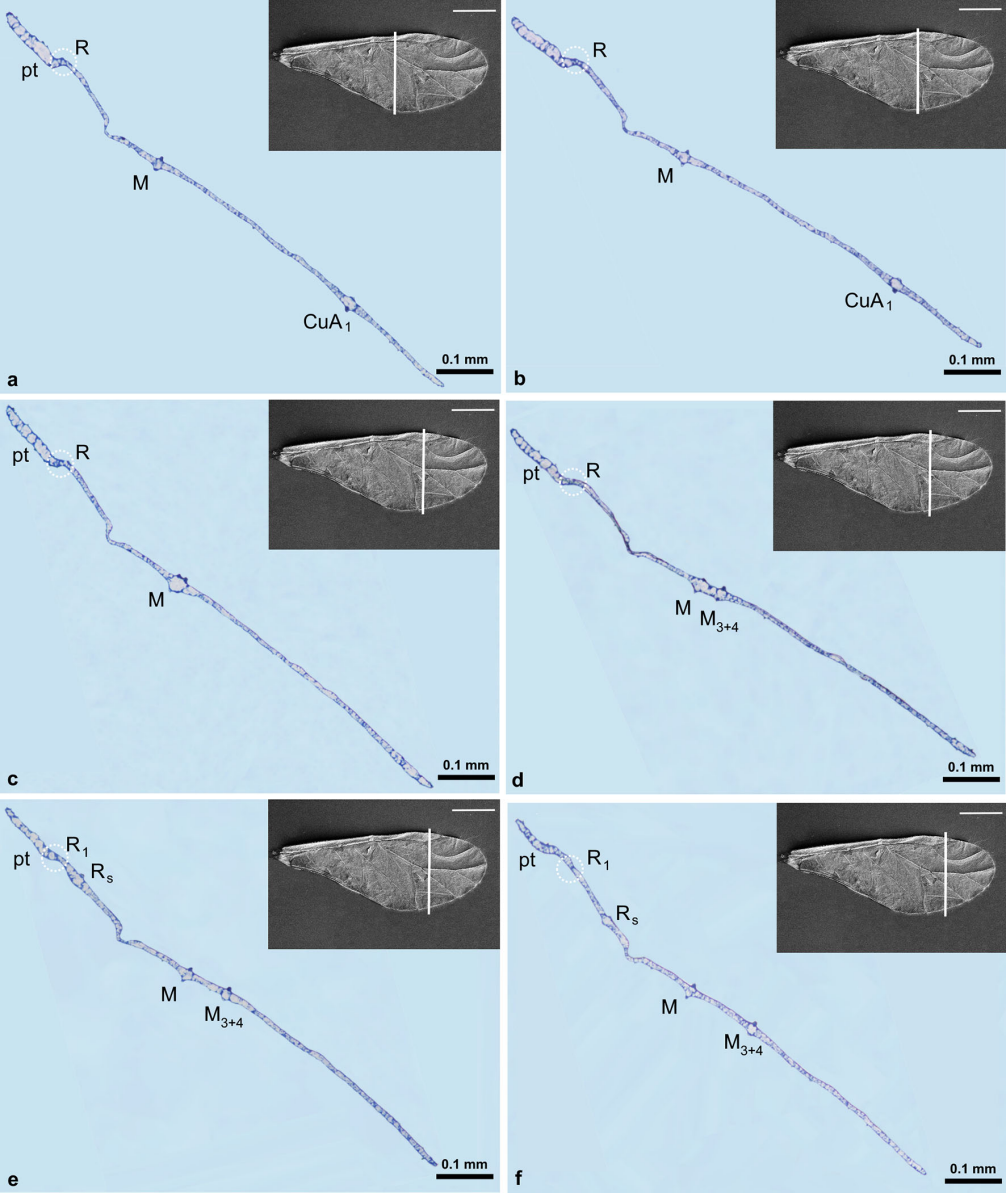
Cross-sections of the forewing of *Aphis fabae* (Scopoli 1763) under magnification **a**–**f** ×10; LM. SEM *scale bar* 0.5 mm

**Fig. 9:**
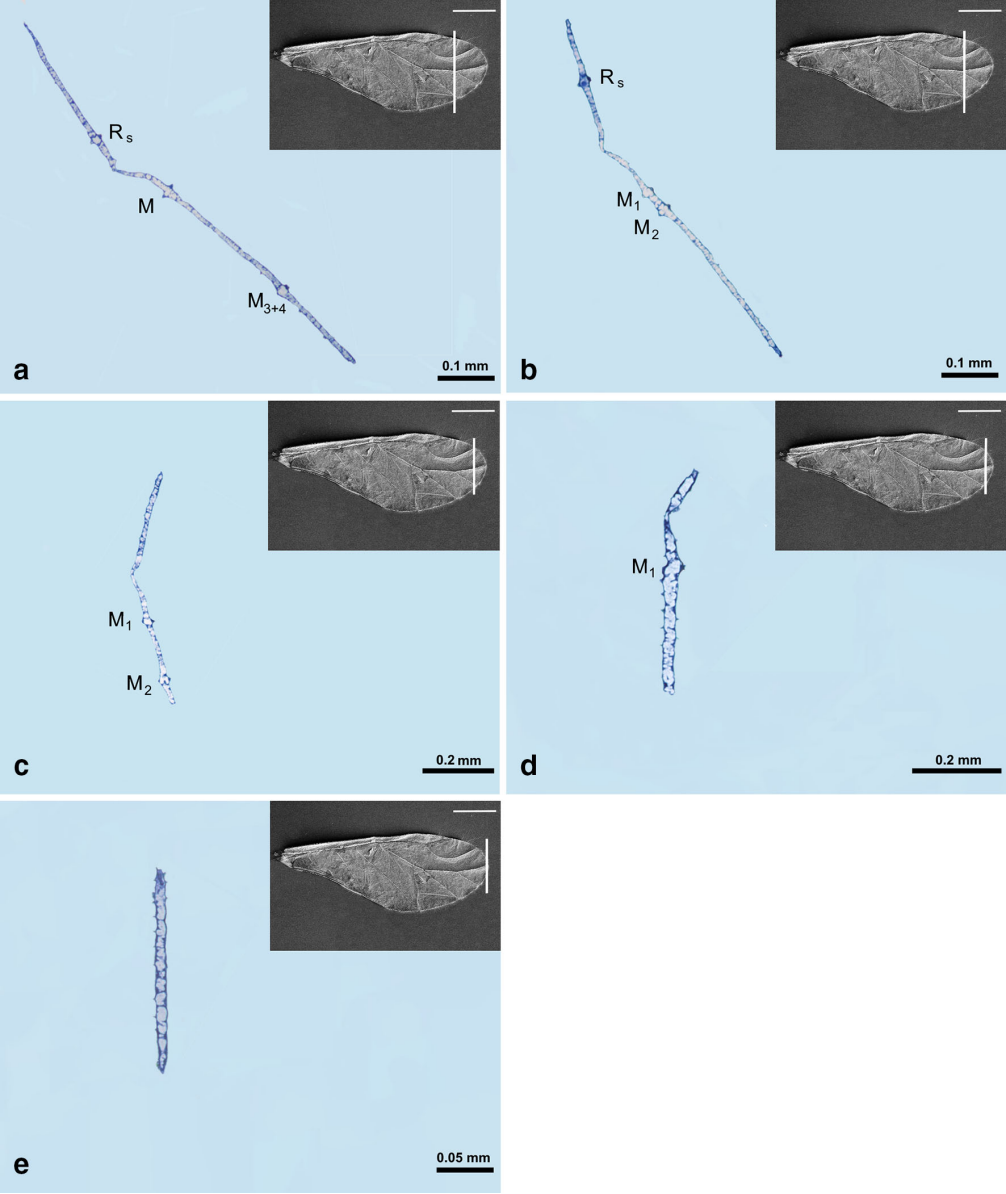
Cross-sections of the forewing of *Aphis fabae* (Scopoli 1763) under magnification **a** and **b** ×10; **c**–**e** ×20; LM. SEM *scale bar* 0.5 mm

The channel for common stem is visible as the main U-shaped indentation of the wing on nine sections (Figs. 6a–d, 7a–e, Fig. 6a white arrow), but its content is changing. All three veins (R + M + CuA) are present in Figs. 6a–d, 7a, b. In Fig. 7c–e, only R + M are building common stem, and from Figs. 7f till 8d, the stem includes only vein R. The latter is divided into R1 and Rs in Fig. 8e, f, but from Fig. 9a only vein Rs is present.

The first three pictures, Fig. 6a–c, show the most basal part of the wing. Around the wing membrane (Fig. 6a), there is a clearly visible cuticle (c) and epidermal cells (e) inside the wing. Costal vein and common stem, consisting of R + M + CuA, are visible.

Media is present on cross-sections from Fig. 7f, as a rounded vein, with small black dots on upper and lower walls. Starting from Fig. 8c, media begins to divide into two separate veins. On upper wall of this vein, there are two black dots. New branch of media, M_3+4_, is presented in Figs. 8d–f and 9a. Next, Figs. 8f and 9b, c, show division of M into M_1_ and M_2_, until the latter are no longer visible. The last section (Fig. 9e) shows only wing membrane, without any vein.

The second branch of cubital vein (CuA_2_) is present in Fig. 7a–c, where the first branch (CuA_1_) is also visible. The latter is marked in Figs. 7c–f, 8a, b.

Pterostigma is present in Fig. 8a–f, as an extension of the wing membrane; it is not entirely empty but seems to contain cells.

Folded anal margin of the wing is visible in Fig. 7a–d (white dotted circle).

A small indentation between R + M visible in Figs. 7e, f, 8, and 9a–c is the artifact created during wing embedding.

## Discussion

The nomenclature of wing veins in insects should differentiate between true veins and (vein-like) false veins. The first group should refer to veins composed of nerves, tracheae, and the cavity for hemolymph. The other should contain internally empty veins, which serve only as reinforcement elements (Dudley 2000). The elements called “wing veins” which are visible on *Aphis fabae* dorsal wing side, are in fact more sclerotized with chitin acting as reinforcement of the veins and the whole wing.

According to Wojciechowski (1992), the lack of costal vein is considered as synapomorphy among aphids and coccids. Patch (1909) claimed that this vein is present in aphids but it has no trachea; no trachea could have been identified in our study either. Moreover, Shcherbakov (2007) stated that the lack of C trachea is regarded as synapomorphy of Aphidomorpha + Coccomorpha. In our ongoing studies, we want to check if costal vein and its trachea are present in coccid representatives.

Detailed studies done by Patch (1909) also showed that there is no such thing as subcostal trachea (Sc_t_), but vein Sc is present, so it is not surprising that we cannot mark trachea of subcostal vein. It seems more interesting that subcostal vein is not shown on any part of the section. The lack of vein Sc and its trachea mentioned by Wojciechowski (1992) as apomorphy in aphids is confirmed in our studies.

At the beginning of sections, common stem is a rounded hole in the wing (Fig. 6a). Further, each of the three tracheae is separated by thin epidermal walls (Fig. 6b), but a little further* R*_*t*_ is separated and Mt + CuA_t_ are enclosed in one “cell”. It looks as if tracheae of M and CuA run together in common stem, independent of* R*_*t*_ (Fig. 6c). Unfortunately, it is visible only on the initial sections—next ones show no trace of trachea. The fusion of veins R + M + CuA in forewings of Sternorrhyncha is evident especially in aphids and psyllids (Nel et al. 2012).

Recently, Szwedo et al. (2015) described the oldest Aphidomorpha species, which showed an uncommon condition among aphids—branch CuA_2_ was thicker than CuA_1_. In our study, two branches of CuA (CuA_1_ and CuA_2_) are widely separated, almost parallel and convex, just as pointed out by Shcherbakov (2007). It is regarded as an apomorphic condition in aphids (Shaposhnikov 1980, 1985, 1987). Based on those conclusions, we can assume that the species described by Szwedo et al. (2015) belong to another, independent developmental line.

In addition, nodal line (transverse flexion line) is not visible in figures from SEM. Moreover, in our SEM study medial vein is convex on its entire course, which is not consistent with Shcherbakov and Wegierek (1991), who stated that M is partly convex–concave to allow upstroke.

R1 is not clearly visible in sections due to the fact that it is a very inconspicuous vein. Besides, it is concave compared to other wing veins on the dorsal side.

Pterostigma is described as a pigmented spot near the end of the wing tip; the element is responsible for increasing speed. In Hemiptera it is present on forewings only. Very little is known about this part of the wing in that insect, especially its inner structure. Generally, it is regarded as blood sinus (Arnold 1963). Our studies showed that, on sections it is a broadened wing membrane where the dorsal and ventral sides are connected by a pattern of chitinous reinforcements in the shape of transverse trabeculae. Being wider than the other parts of the wing, it can really play a role as an element for better wing-flapping performance without additional energy (Norberg 1972).

During the course of their evolution, aphids tend to diminish their body size and reduce the claval fold and anal region of the wings (Shaposhnikov 1980, 1985, 1987; Shcherbakov and Wegierek 1991; Shcherbakov 2007). Accordingly, such structures do not exist in extant aphids. Shcherbakov (2007) also indicated the presence of submarginal claval veins PCu + 1A, in aphids’ and coccids’ forewings. Cross-sections presented here do not confirm their existence. Probably those two veins moved so close to the marginal part of the anal margin of the wing (Fig. 7a–d) that they have become a part of wing-coupling apparatus in aphids, linking both wing pairs during the flight. The coupling apparatus consists of the fold of the forewing and the hamuli of the hindwing; the latter may vary in number in different species (Ni et al. 2002). In addition, Szwedo et al. (2015) claimed that PCu and A_1_ were present in the oldest Aphidomorpha known so far (from the Middle Permian). Probably, these veins are not preserved in recent aphids. The same authors indicated the presence of subcostal posterior vein (ScP) near the costal margin of the forewing of aphid’s ancestor. We have not found any evidence to confirm that in the examined aphis.

The present examination identified the following features on *A. fabae* forewings: the presence of costal vein without its trachea; lack of subcostal vein; common stem of radius, media, and cubital anterior veins with tracheae in one tunnel at the beginning; widely separated, almost parallel and convex cubital anterior veins; pterostigma as a broadened part of the wing covered by scale-like elements; vein R1 concave on the dorsal side, while all other veins are convex; lack of anal fold and nodal line. It is also important that veins do not reach the wing apex and are not connected with the common stem.

Further investigations are required to supplement the results with the remaining representatives of Sternorrhyncha suborder (studies in process). After that, we will be able to verify current nomenclature of wing veins among these insects and decide which wing veins can be called true and which ones vein-like (false).
